# Is it stress? The role of stress related systems in chronic food restriction-induced augmentation of heroin seeking in the rat

**DOI:** 10.3389/fnins.2013.00098

**Published:** 2013-06-06

**Authors:** Firas Sedki, Zarish Abbas, Staci Angelis, Jeffrey Martin, Tracey D'Cunha, Uri Shalev

**Affiliations:** Department of Psychology, Center for Studies in Behavioral Neurobiology/Groupe de Recherche en Neurobiologie Comportementale, Concordia UniversityMontreal, QC, Canada

**Keywords:** self-administration, chronic food restriction, corticotropin-releasing factor, corticosterone, adrenalectomy, R121919, α-helical CRF, RU486

## Abstract

Drug addiction is a chronic disease characterized by recurring episodes of abstinence and relapse. The precise mechanisms underlying this pattern are yet to be elucidated, but stress is thought to be a major factor in relapse. Recently, we reported that rats under withdrawal and exposed to a mild chronic stressor, prolonged food restriction, show increased heroin seeking compared to sated controls. Previous studies demonstrated a critical role for corticotropin-releasing factor (CRF) and corticosterone, hormones involved in the stress response, in acute food deprivation-induced reinstatement of extinguished drug seeking. However, the role of CRF and corticosterone in chronic food restriction-induced augmentation of drug seeking remains unknown. Here, male Long-Evans rats were trained to self-administer heroin for 10 days in operant conditioning chambers. Rats were then removed from the training chambers, and subjected to 14 days of unrestricted (sated rats) or a mildly restricted (FDR rats) access to food, which maintained their body weight (BW) at 90% of their baseline weight. On day 14, different groups of rats were administered a selective CRF_1_ receptor antagonist (R121919; 0.0, 20.0 mg/kg; s.c.), a non-selective CRF receptor antagonist (α-helical CRF; 0.0, 10.0, 25.0 μg/rat; i.c.v.) or a glucocorticoid receptor antagonist (RU486; 0.0, 30.0 mg/kg; i.p.), and underwent a 1 h drug seeking test under extinction conditions. An additional group of rats was tested following adrenalectomy. All FDR rats showed a statistically significant increase in heroin seeking compared to the sated rats. No statistically significant effects for treatment with α-helical CRF, R121919, RU486 or adrenalectomy were observed. These findings suggest that stress may not be a critical factor in the augmentation of heroin seeking in food-restricted rats.

## Introduction

Stress is consistently reported by drug users as a factor in subjective craving as well as in the initiation, maintenance, and relapse of drug use (Brewer et al., 1998; Matheny and Weatherman, 1998; Sinha and O'Malley, 1999; Sinha, 2001, 2008). A role for stress in triggering relapse to drugs has also been identified in retrospective studies in which interviews and questionnaires were given to addicts, as well as in controlled laboratory studies (Kosten et al., 1983, 1986; Sinha et al., 2006). Of many potential stressful life events, a particularly interesting one is exposure to caloric restriction, which precipitates negative affective states, as well as physiological changes including increases in circulating glucocorticoid levels (Dallman et al., 1999; Tomiyama et al., 2010). Studies showing an increased risk for relapse among calorically restricted abstinent smokers (Hall et al., 1992), and a positive correlation between the severity of diet and the risk of drug taking in young women (Krahn et al., 1992), suggest a strong link between caloric restriction and drug intake. Moreover, in humans, only prolonged food restriction, and not acute food deprivation, is associated with increased drug taking (Zacny and de Wit, 1991; Cheskin et al., 2005, also see D'Cunha et al., 2013).

In laboratory animals, the effects of restricting food availability on drug-associated behaviors have been demonstrated unequivocally. The initiation and maintenance of drug intake are reliably enhanced following periods of caloric restriction (Piazza and Le Moal, 1998; Lu et al., 2003). Acute food deprivation (FD: 24–48 h), can induce reinstatement to drug seeking in rats with a history of heroin or cocaine self-administration (Shalev et al., 2000, 2003). This effect is attenuated by antagonism of the stress neuropeptide corticotropin-releasing factor (CRF) receptor, but not the removal of corticosterone (Shalev et al., 2003, 2006), a pattern of results similar to those obtained by Shaham et al. (1997) in a study utilizing footshock as a stressor.

Interestingly, although both acute FD and chronic food restriction (days to weeks) decrease BW and augment drug seeking in rodents, they can have quite different metabolic and behavioral effects on the organism. For example, Fulton et al. (2000) demonstrated a decreased threshold for electrical brain stimulation reward in chronically food restricted but not acutely food deprived rats. Similarly, increased cigarette smoking following prolonged food restriction, but not acute food deprivation, has been reported in human subjects (Zacny and de Wit, 1992; Cheskin et al., 2005). In response to these findings, we have recently developed a clinically relevant model, in which drug seeking in drug-free rats with a history of heroin self-administration is tested following prolonged food restriction. Using this model, our laboratory has reported a dramatic enhancement of heroin seeking in food restricted rats under going withdrawal (>250%) compared to sated controls (D'Cunha et al., 2013).

Although the extrahypothalamic CRF system is critically involved in acute FD-induced reinstatement of heroin seeking (Shalev et al., 2006), its role in chronic food restriction-induced augmentation of drug seeking is not known. In addition, while a key role for a stress-induced increase in corticosterone in acute FD-induced reinstatement was ruled out (Shalev et al., 2003, 2006), previous studies demonstrated that pharmacological blockade of corticosterone synthesis or complete removal of corticosterone attenuates chronic food restriction-induced augmentation of drug-related behaviors (Deroche et al., 1995; Marinelli et al., 1996). Thus, the aim of this study was to investigate the involvement of CRF and corticosterone, in the augmentation of heroin seeking following prolonged food restriction in rats under withdrawal.

## Materials and methods

### Subjects

One hundred and thirty-three male, Long-Evans rats (Charles River, St. Constant, Quebec, Canada; 300–350 g) were used. Before surgery, animals were pair-housed for 1 week in the animal care facility (ACF) under reverse light/dark conditions (lights OFF at 0930). Following intravenous (IV) catheterization, and 2 days of recovery, rats were single-housed in plastic shoebox cages before being transferred to operant conditioning chambers for drug self-administration. Following self-administration training, rats were returned to the ACF and single-housed in shoebox cages for the drug withdrawal phase. Except for the withdrawal phase, all rats were given unrestricted access to food and water. Rats were treated according to the Canadian Council on Animal Care guidelines, and approval was granted by the Concordia University Animal Research Ethics Committee.

### Surgical procedures

#### Intravenous catheterization and intracerebroventricular (i.c.v.) cannulation

Rats were implanted with IV silastic catheters (Dow Corning, Midland, MI, USA) under xylazine/ketamine (13.0 + 90.0 mg/kg; i.p.). Three centimeters of silastic catheter was inserted through a small incision on the right jugular vein, and secured using silk sutures. The remainder of the catheter was passed subcutaneously to the skull, attached to a modified 22-gauge cannula (Plastics One, Roanoke, VA) and anchored to the skull using dental cement and 5 jeweler's screws. Some rats (Experiment 1A) were also implanted with a 22-gauge guide cannula (Plastics One) aimed 2 mm above the right or left lateral ventricle (AP, ±0.5; ML, +1.4; DV, −3.0; relative to bregma) to allow for i.c.v. injections.

Following surgery, rats were administered buprenorphine (600.0 μg/rat; s.c.; Schering-Plough Ltd., Welwyn Garden City Hertfordshire, UK) and penicillin (450,000 IU/rat; s.c.) to reduce pain and prevent infection. Catheters were flushed daily with heparin/gentamicin (7.5 IU +12.0 mg/rat) to prevent blockage and infection.

#### Adrenalectomy

Bilateral adrenalectomy (ADX) surgeries were performed on withdrawal day 10 on rats in Experiment 2B, under isoflurane USP anesthetic. The adrenal glands were rapidly removed via the dorsal approach, and the rats were given ketoprofen injections (5.0 mg/kg; s.c.) following surgery to reduce pain. Sham-operated rats were exposed to the same procedure as the ADX rats, with the exception that the adrenal glands were not removed. After surgery, the ADX rats were given physiological saline (0.9% NaCl) in their drinking bottles.

### Apparatus

Rats were housed individually in operant conditioning chambers (Coulbourn Instruments, Allentown, PA, USA; 29.0 × 29.0 × 25.5 cm) enclosed in sound attenuating wooden compartments equipped with a fan. Each chamber was fitted with two retractable levers (Coulbourn Instruments) mounted 9 cm above the floor of the right sidewall. Responses on the “active” lever activated the infusion pump and a cue-light/tone (Coulbourn Instruments, Sonalert, 2.9 KHz) located above the lever. Responses on the “inactive” lever had no programmable consequences. Rats were attached to the infusion pump via a liquid swivel (Instec Laboratories Inc., Boulder, CO, USA) and Tygon tubing shielded with a metal spring.

### Drugs

Heroin HCl (a contribution from the National Institute for Drug Abuse, Research Triangle Park, NC, USA) was dissolved in sterile saline (5.0 mg/ml) and then further diluted with saline, for each rat according to BW to yield 0.1 mg/kg/infusion.

R121919, the selective CRF_1_ receptor antagonist, was kindly supplied by Dr. Kenner Rice (National Institute on Drug Abuse, NIH, Baltimore, MD, USA). R121919 was dissolved in a 20% β-Cyclodextrin (Sigma-Aldrich) sterile saline solution to a concentration of 10.0 mg/ml and adjusted to a pH of 4.5. The antagonist was injected (s.c.) at a final dose of 20.0 mg/kg. A solution of 20% β-Cyclodextrin mixed in sterile saline solution was used as a vehicle injected at a volume of 2.0 ml/kg. A similar dose of R121919 reduced both drug self-administration and anxiety-like behaviors (Greenwell et al., 2009; Gutman et al., 2010).

The non-selective CRF antagonist, α-helical CRF (Sigma-Aldrich), was dissolved in sterile water to a concentration of 5.0 μg/μl or 12.5 μg/μl. α-helical CRF was injected i.c.v. over 2 min at a rate of 1.0 μl/min for a final dose of 10.0 or 25.0 μg/rat. Sterile water was used as a vehicle. Injections were made using a syringe pump (Harvard Apparatus, Holliston, MA, USA) connected to a 10 μl Hamilton syringe. This syringe was attached via polyethylene-20 tubing to a 28-gauge injector (Plastics One) that extended 2 mm below the guide cannulae. The injector was kept in place for 1 min after the injections to allow for proper diffusion of the drug. The doses for α-helical CRF were based on previous reports (Baldwin et al., 1991; Krahn et al., 1986; Shalev et al., 2006).

RU486, a glucocorticoid receptor antagonist, was dissolved using a 25% β-Cyclodextrin (Sigma-Aldrich), 1% Tween-80 (Sigma-Aldrich), 2–3 drops of 1N HCl, and sterile water mixture which also served as the vehicle. RU486 and vehicle solutions were adjusted to a pH of ~5.6 and injected (i.p.) at a dose of 30 mg/kg (RU486), or a volume of 1 ml/kg (vehicle). A similar dose was shown to attenuate stress-induced reinstatement of alcohol seeking (Simms et al., 2012).

### Procedure

#### Self-administration

Following a 24-h habituation period in the chamber, rats were trained to self-administer heroin in daily three 3-h sessions separated by 3-h intervals for 10 days. The first daily session began shortly after the onset of the dark phase with the extension of the active and inactive levers into the conditioning chamber, illumination of a house-light and activation of the cue-light/tone complex for 30 s. Responses on the active lever, which was armed with a fixed ratio-1 schedule (FR-1), resulted in activation of the drug pump (5 s, 0.13 ml/infusion) and the initiation of a 20 s timeout during which the house-light was turned off and the cue light/tone complex above the active lever was activated. During the timeout period, active lever responses were recorded but not reinforced. Following each 3-h session, the active lever was retracted whereas the inactive lever was not retracted until 1 h before the first session of the following day. Inactive lever responses were recorded but had no programmable consequences.

### Drug withdrawal phase

#### Experiments 1A, 1B, and 2A

Following self-administration training, rats were individually housed in the ACF, and given unrestricted access to food and water for one drug-washout day. Rats were then divided into two groups: food restricted (FDR) or Sated that were matched according to BW, number of infusions, and active lever responses across the last 5 days of training. Following the washout day, FDR rats had their food removed and were fed ~15 g of rat chow at 1330. The amount of food was adjusted through 14 days of food restriction to maintain the food restricted rats' BW at ~75–80% of the Sated rats and 90% of their baseline BW.

#### Experiment 2B

Rats were treated as in Experiment 2A, except for the ADX surgery that was performed on day 10 of withdrawal. Following surgery, rats were given 25 g of food and allowed 1 day of recovery, after which they were re-restricted to the previously described regimen and allowed 2 extra days of withdrawal to ensure their BWs reached criteria.

### Drug-seeking test

#### Experiment 1A: the effect of treatment with the selective CRF_1_ receptor antagonist, R121919

On withdrawal day 14, rats were returned to the operant conditioning chambers and attached to the metal spring. The drug-seeking test consisted of a 1-h session during which active lever responses had the same consequences as in training excluding the availability of the drug. A subcutaneous injection of R121919 (0.0, 20.0 mg/kg, s.c.) was administered 30 min before the test session.

#### Experiment 1B: the effects of treatment with the non-selective CRF receptor antagonist, α-helical CRF, on chronic food restriction-induced augmentation of heroin seeking in the rat

The testing procedure was similar to the one described for Experiment 1A, except that the rats were given an ICV injection of α-helical CRF (0.0, 10.0 or 25.0 μg/rat, i.c.v.) 10 min before the test session.

#### Experiment 1C: the effects of treatment with the non-selective CRF receptor antagonist, α-helical CRF, on open-field behavior

This experiment was designed to verify the efficiency of the α-helical CRF treatment used in Experiment 1B. Previously, α-helical CRF has been shown to have anxiolytic properties (Koob and Heinrichs, 1999). To ensure that the antagonist had similar effects in our hands, eight rats that participated in Experiment 1B were given a test for anxiety. These rats were food restricted for an additional 8 days following the drug-seeking test and on day 8 at 1330, were brought into a novel, brightly lit, environment and placed in a white circular arena (diameter 137 cm; height 46 cm) with one food pellet in the center. Rats were placed at either the north, south, west, or east positions of the arena and allowed to explore the environment for 10 min. The rats' behavior was recorded by a video camera. Two variables were then scored from the video recordings. The first, latency to consumption, was defined as the time for the rat to first consume a portion of the food pellet. The second, number of approaches, was defined as the number of times a rat approached the food pellet until first consumption.

#### Experiment 2A: the effect of treatment with the glucocorticoid receptor antagonist, RU486

Testing procedure was similar to the one described for Experiment 1A, except that the rats were given an injection of RU486 (30.0 mg/kg, i.p.) or vehicle in the ACF 45 min before the test session.

#### Experiment 2B: the effect of ADX

Testing procedure was similar to the one described for Experiment 1A, except that, other then the ADX surgery, no further treatment was given before the test.

#### Plasma corticosterone determination

Immediately following the drug-seeking test (1030), tail blood was collected, and plasma was separated by centrifugation at 10,000 rpm for 10 min. Samples were stored at −80°C. Plasma samples were analyzed for corticosterone levels using a corticosterone specific enzyme-linked immunosorbent assay (ELISA) kit (Enzo Life Sciences: Cedarlane, Burlington, ON, Canada). The reported detection sensitivity for the kit is 27.0 pg/ml.

### Statistical analysis

All analyses were conducted using SPSS software (IBM, SPSS Statistics, version 20). Training data for all rats were analyzed using a repeated measures ANOVA, with training day (1–10) as a within-subjects factor and the number of active lever responses, inactive lever responses or number of infusions as the dependent variables.

#### Experiment 1

Number of responses on the active and inactive levers during the drug-seeking test sessions were analyzed separately using Two-Way ANOVAs. Antagonist dose (Experiment 1A: 0.0, 20.0 mg/kg; Experiment 1B: 0.0, 10.0, 25.0 μg/rat) and feeding condition (FDR, Sated) served as between subject factors.

The effect of treatment with α-helical CRF (0.0, 25.0 μg/rat) on the open field behavior was analyzed using two independent samples *t*-tests. Latency to consumption and number of approaches were the dependent variables.

#### Experiment 2

Number of responses on the active and inactive levers during the test session were analyzed separately using Two-Way ANOVAs. Antagonist dose (Experiment 2A: 0.0, 30.0 mg/kg) or surgery condition (Experiment 2B: ADX, Sham) and feeding condition (FDR, Sated) served as the between subject factors.

Plasma corticosterone levels (ng/ml), sampled following the test sessions in Experiment 2, were analyzed using a Two-Way ANOVA. Antagonist dose (Experiment 2A: 0.0, 30.0 mg/kg) or surgery condition (Experiment 2B: ADX, Sham) and feeding condition (FDR, Sated) served as the between subject factors.

In all analyses, statistically significant interactions were investigated with the appropriate *post-hoc* tests and the critical threshold for statistically significant results was set at *p* ≤ 0.05.

## Results

All rats acquired reliable heroin self-administration behavior. Mean ± SEM number of infusions and number of active and inactive lever responses made on the last day of heroin self-administration training, for each experiment, are shown in Table 1. There were no statistically significant differences in any of the above parameters between the different experimental groups within each experiment.

**Table 1:** **Mean ± SEM number of heroin infusions and active and inactive lever responses made on the last day of training (9 h) in each experiment**.

	**Infusions**	**Active lever**	**Inactive lever**
Experiment 1A	42.08 ± 4.25	155.72 ± 18.97	13.28 ± 3.36
Experiment 1B	35.06 ± 1.91	99.62 ± 13.40	6.90 ± 0.84
Experiment 2A	40.23 ± 4.60	141.62 ± 23.45	18.00 ± 8.27
Experiment 2B	36.72 ± 2.84	116.66 ± 17.77	25.03 ± 6.01

### Experiment 1A: the effects of treatment with the selective CRF_1_ receptor antagonist, R121919, on chronic food restriction-induced augmentation of heroin seeking in the rat

Five rats were removed due to catheter leakage, failure to train, or detached head-cap. Thus, the final analysis included 25 rats in 4 experimental groups: FDR-0.0 (*n* = 5), FDR-20.0 (*n* = 7), Sated-0.0 (*n* = 6), Sated-20.0 (*n* = 7). On the test day, average BW of the rats in the Sated group (*n* = 13; 452.92 ± 9.65 g) was statistically significantly greater than that of the rats in the FDR group (*n* = 12; 333.50 ± 6.47 g; *t*_(23)_ = −10.12, *p* < 0.001).

**Figure 1:**
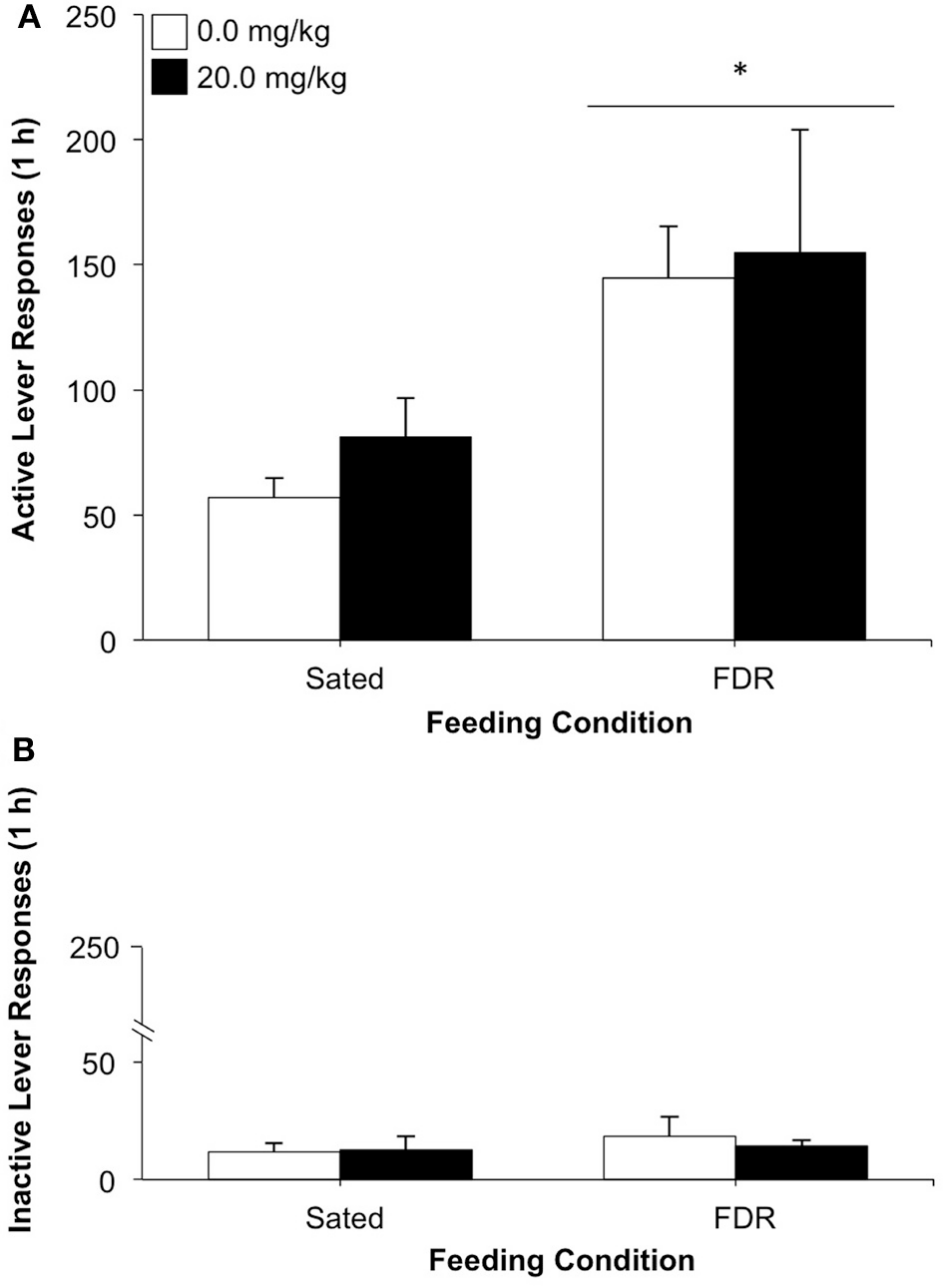
**The effect of treatment with the selective CRF_1_ receptor antagonist, R121919 (0.0, 20.0 mg/kg, s.c.) on heroin seeking in food-restricted (FDR) and sated rats under withdrawal; Experiment 1A.** Data shown are the mean (+SEM) numbers of active **(A)** and inactive **(B)** lever responses made on test day by rats in the FDR (*n*'s: FDR-0.0 = 6, FDR-20.0 = 7) and Sated (*n*'s: Sated-0.0 = 5, Sated-20.0 = 7) groups. Test day consisted of one 1-h drug-seeking session under extinction conditions, following heroin self-administration training and 14 days of withdrawal under FDR or sated conditions. ^*^Significantly different from the sated condition, *p* = 0.016.

The mean number of active lever responses performed by the rats in the FDR group was almost three times higher than the lever responses made by the Sated group during the drug-seeking test. The robust effect was confirmed by a statistically significant main effect of feeding condition [*F*_(1, 21)_ = 6.91, *p* = 0.016, η^2^ = 0.24; Figure 1A]. No statistically significant effects were found for antagonist dose or for the interaction feeding condition × antagonist dose. No statistically significant effects were observed for inactive lever responding on the test day (Figure 1B).

### Experiment 1B: the effects of treatment with the non-selective CRF receptor antagonist, α-helical CRF, on chronic food restriction-induced augmentation of heroin seeking in the rat

Eight rats were removed due to catheter leakage, failure to train or detached head-caps. Therefore, the final analysis included 52 rats in 6 experimental groups: FDR-0.0 (*n* = 8), FDR-10.0 (*n* = 11), FDR-25.0 (*n* = 7), Sated-0.0 (*n* = 10), Sated-10.0 (*n* = 11), Sated-25.0 (*n* = 5). On test day, the average BWs of rats in the Sated group (*n* = 26; 439.62 ± 9.27 g) was statistically significantly greater than that of rats in the FDR groups (*n* = 26; 333.08 ± 4.17 g; *t*_(50)_ = −10.48, *p* < 0.001).

**Figure 2:**
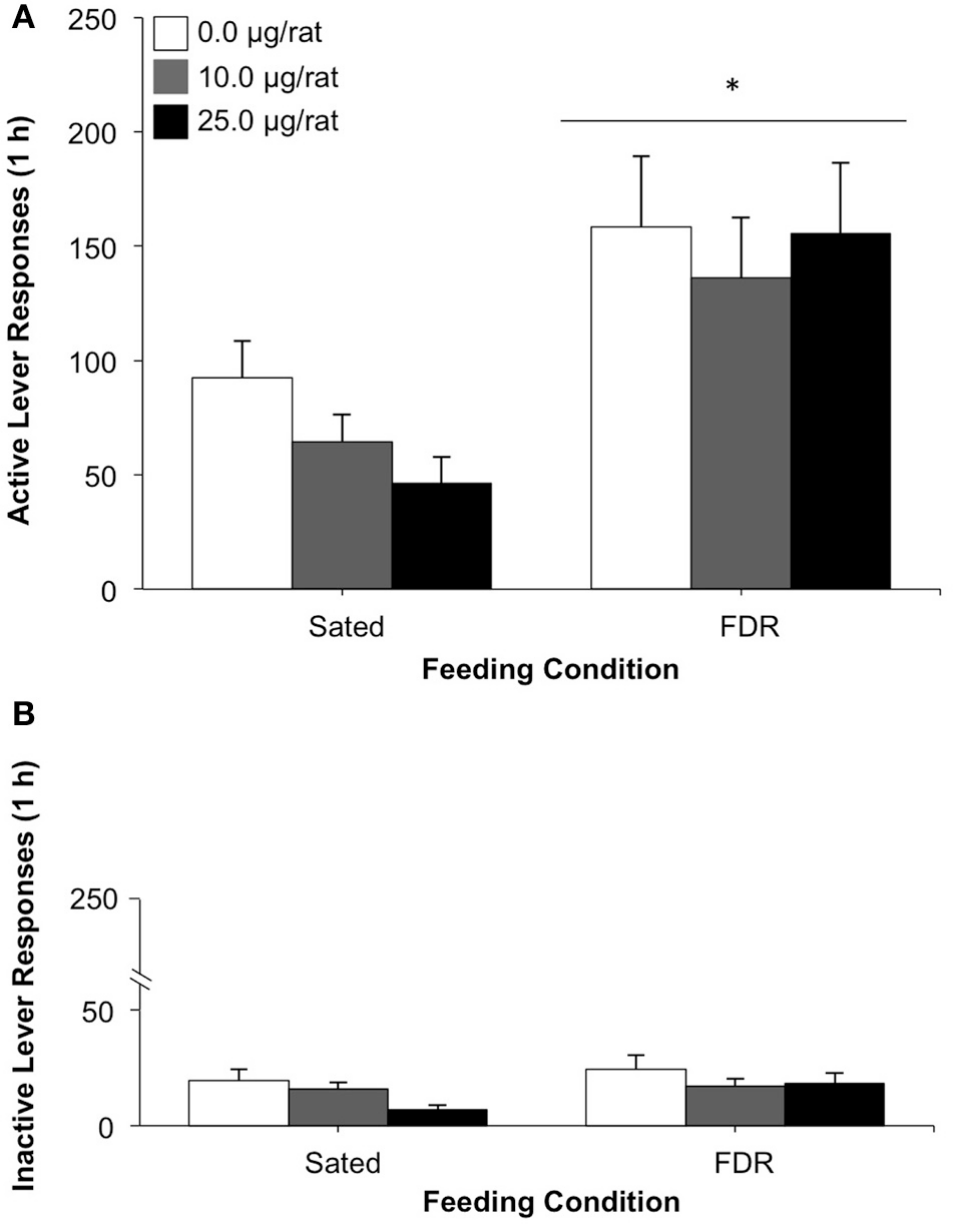
**The effect of treatment with the non-specific CRF receptor antagonist, α-Helical CRF (0.0, 10.0, 25.0 μg/rat, i.c.v.) on heroin seeking in food-restricted (FDR) and sated rats under withdrawal; Experiment 1B.** Data shown are the mean (+SEM) numbers of active **(A)** and inactive **(B)** lever responses made on test day by rats in the FDR (*n*'s: FDR-0.0 = 8, FDR-10.0 = 11, FDR-25.0 = 7) and Sated (*n*'s: Sated-0.0 = 10, Sated-10.0 = 11, Sated-20.0 = 5) groups. Test day consisted of one 1-h drug-seeking session under extinction conditions, following heroin self-administration training and 14 days of withdrawal under FDR or sated conditions. ^*^Significantly different from the sated condition, *p* < 0.001.

Rats in the FDR group made a higher number of responses on the active lever during the drug-seeking test, compared to the Sated group (see Figure 2A). This finding was supported by a statistically significant main effect of feeding condition, [*F*_(1, 46)_ = 17.68, *p* < 0.001, η^2^ = 0.27]. No statistically significant effects were found for either the antagonist dose or for the feeding condition × antagonist dose interaction. No significant effects were observed for inactive lever responding on test day (Figure 2B).

### Experiment 1C: the effect of treatment with the non-selective CRF receptor antagonist, α-helical CRF on open-field behavior

**Figure 3:**
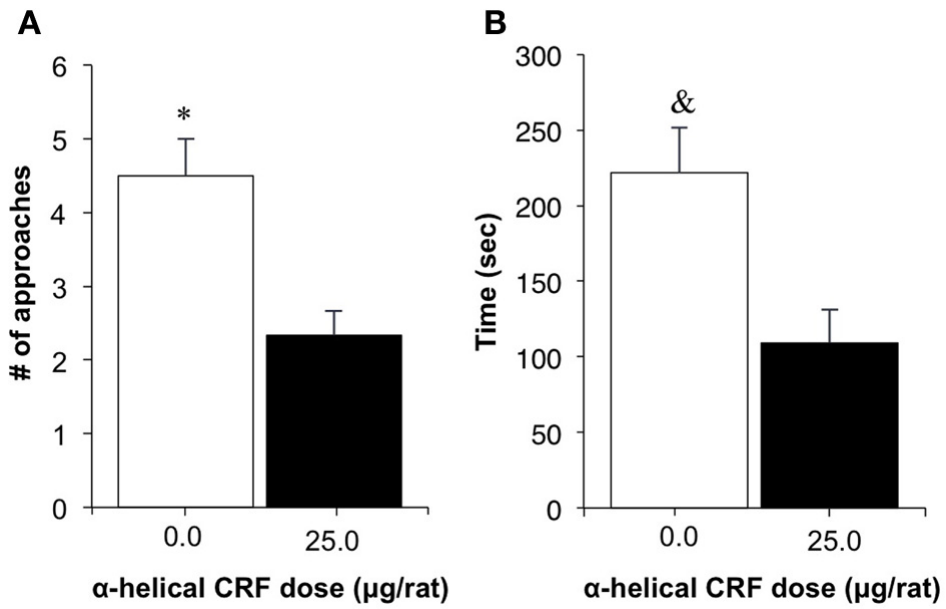
**The effect of treatment with the non-specific CRF receptor antagonist α-Helical CRF (0.0, 25.0 μg/rat, i.c.v.) on behavior in an open field, following an 8-day food restriction period; Experiment 1C.** Data shown are the mean (+SEM) latencies to first consumption of **(A)** and number of approaches to **(B)** a food pellet placed at the center of an open arena following injections of α-Helical CRF in food restricted rats (*n*'s: 0.0 = 4, 25.0 = 3). ^*^p = 0.038; & *p* = 0.021.

Rats in the α-helical CRF-treated group demonstrated a statistically significant decrease in latency to first food consumption and the number of approaches prior to first consumption, compared to vehicle controls [*t*_(5)_ = 2.80, *p* = 0.038; Figure 3A; *t*_(5)_ = 3.13, *p* = 0.021; Figure 3B, respectively].

### Experiment 2A: the effect of treatment with the glucocorticoid receptor antagonist, RU486, on chronic food restriction-induced augmentation of heroin seeking in the rat

Three rats were removed due to catheter leakage, failure to train or being an outlier. Therefore, the final analysis included 27 rats in 4 experimental groups: FDR-0.0 (*n* = 7), FDR-30.0 (*n* = 7), Sated-0.0 (*n* = 8), Sated-30.0 (*n* = 5). On test day, average BW of the rats in the Sated group (*n* = 13; 426.23 ± 8.02 g) was statistically significantly greater than that of the rats in the FDR group (*n* = 14; 317.36 ± 5.21 g; *t*_(25)_ = −11.54, *p* < 0.001).

**Figure 4:**
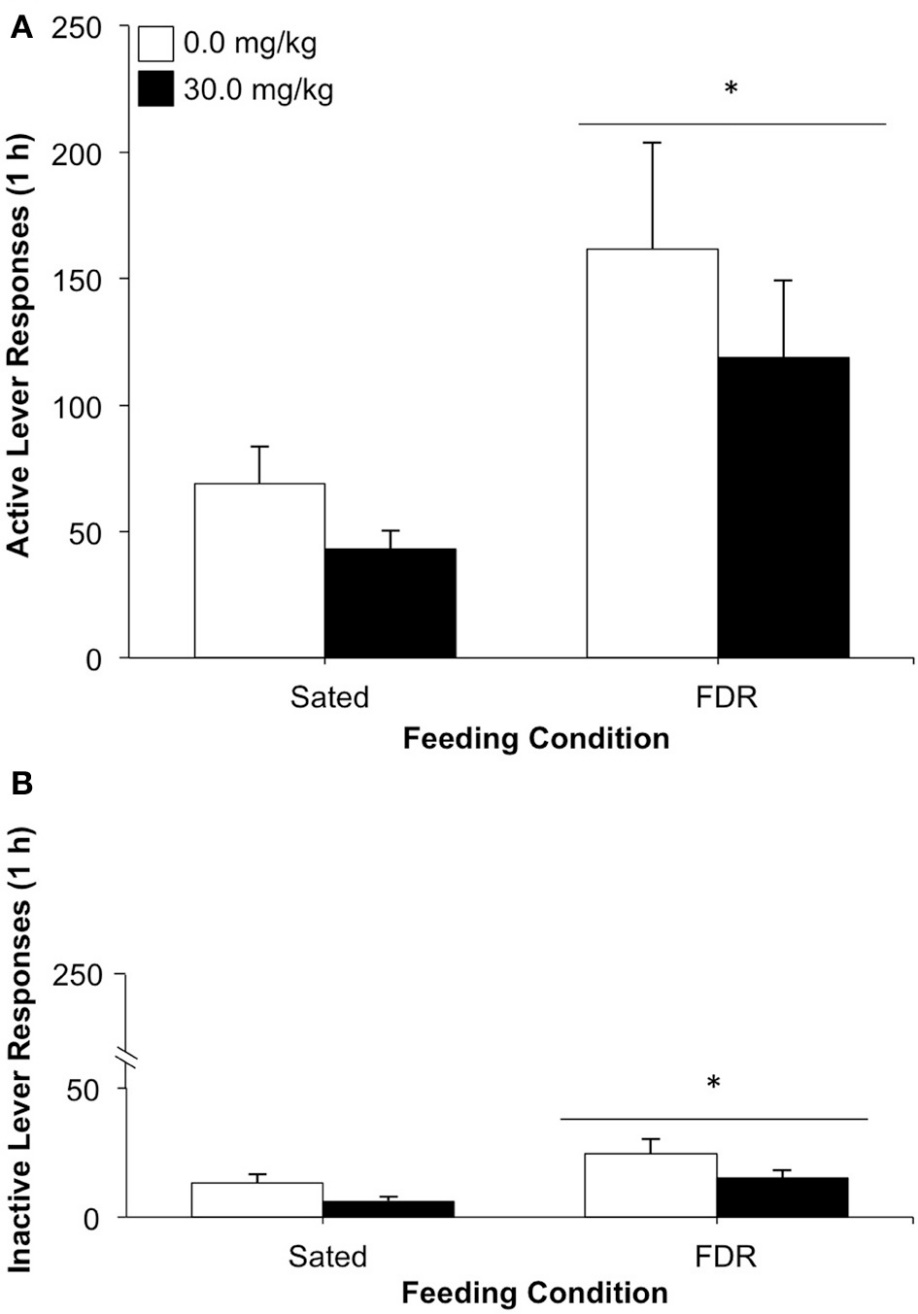
**The effect of treatment with the glucocorticoid receptor antagonist, RU486 (0.0, 30.0 mg/kg, i.p.) on heroin seeking in food-restricted (FDR) and sated rats under withdrawal; Experiment 2A.** Data shown are the mean (+SEM) numbers of active **(A)** and inactive **(B)** lever responses made on test day by rats in the FDR (*n*'s: FDR-0.0 = 7, FDR-30.0 = 7) and Sated (*n*'s: Sated-0.0 = 8, Sated-30.0 = 5) groups. Test day consisted of one 1-h drug-seeking session under extinction conditions, following heroin self-administration training and 16 days of withdrawal under FDR or sated conditions. ^*^Significantly different from the sated condition, *p* < 0.02.

As can be seen in Figure 4A, the FDR group showed more active lever responding than the Sated group, [feeding condition effect: *F*_(1, 23)_ = 8.46, *p* = 0.008, η^2^ = 0.26] during the drug-seeking test. No statistically significant effects for antagonist dose or feeding condition × antagonist dose interaction were observed. The FDR group also responded more on the inactive lever than the Sated group [feeding condition effect: *F*_(1, 23)_ = 6.42, *p* = 0.019, η^2^ = 0.19; Figure 4B]. However, the number of inactive lever responses was very low (<25) and the rats clearly preferred the active lever.

**Figure 5:**
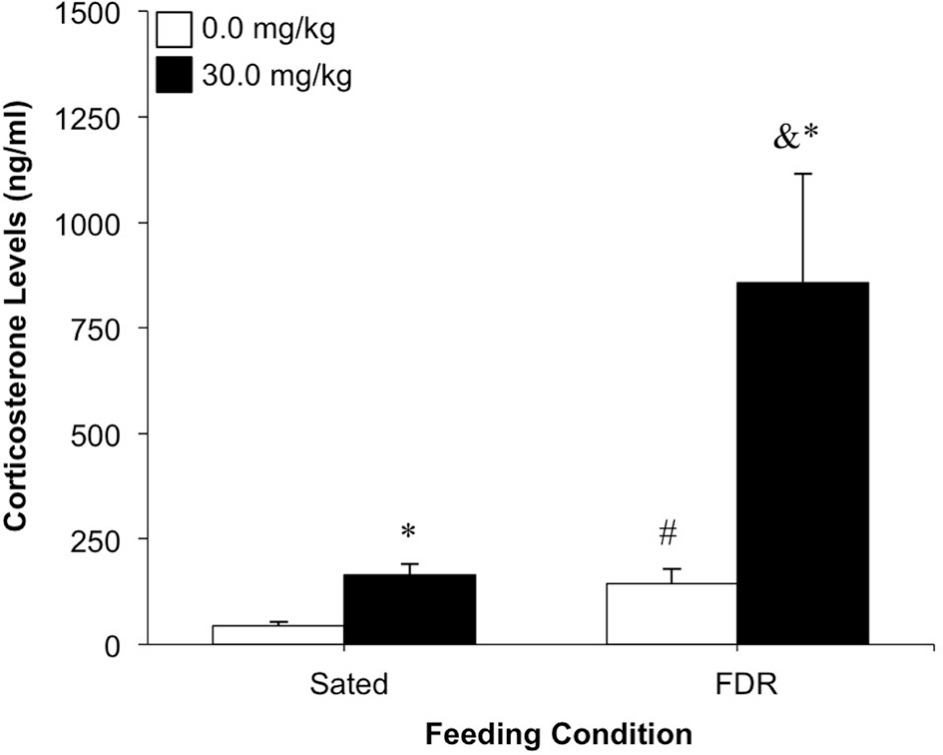
**The effect of treatment with the glucocorticoid receptor antagonist, RU486 (0.0, 30.0 mg/kg, i.p.) on corticosterone levels; Experiment 2A.** Data shown are the mean (+SEM) corticosterone levels (ng/ml) immediately following a drug-seeking test session under extinction conditions, in food restricted rats (*n*'s: 0.0 = 6, 30.0 = 3) and Sated rats (*n*'s: 0.0 = 6, 30.0 = 4). ^*^Significantly different from the 0.0 mg/kg group, *p* < 0.020; ^#^Significantly different from the Sated-0.0 group, *p* = 0.03; & *p* = 0.07 compared to the Sated-30.0 group.

Some corticosterone samples that were collected immediately following the test session were not included in the final analysis due to an unacceptable variability between the duplicates in the ELISA kit (>30%). Administration of RU486 resulted in increased levels of corticosterone in both the sated and FDR rats. However, treatment with RU486 resulted in considerably higher corticosterone levels in the FDR group (Figure 5). ANOVA revealed statistically significant main effects of feeding condition [*F*_(1, 15)_ = 12.80, *p* = 0.003, η^2^ = 0.30] and antagonist dose [*F*_(1, 15)_ = 14.13, *p* = 0.002, η^2^ = 0.34], and a significant feeding condition × antagonist dose interaction effect [*F*_(1, 15)_ = 7.15, *p* = 0.017, η^2^ = 0.17]. Although this significant interaction effect was clearly driven by the dramatic increase in corticosterone levels induced by the RU486 treatment in the FDR rats, corticosterone levels in this group were not statistically significantly different from the sated, RU486-treated rats [*t*_(5)_ = 2.33, *p* = 0.07, Cohen's *d* = 2.08], probably due to the sample size in the FDR, RU486-treated group (*n* = 3). Further *post-hoc* tests are described in Figure 5.

### Experiment 2B: the effect of adrenalectomy on chronic food restriction-induced augmentation of heroin seeking in the rat

Three rats were removed due to catheter leakage, failure to train or detached head-caps. Thus, the final analysis included 29 rats in 4 experimental groups: FDR-Sham = 6, FDR-ADX = 7, Sated-Sham = 7, Sated-ADX = 9.

On test day, the average BW of the Sated group (Sham: 391.57 ± 10.41 g; ADX: 396.89 ± 4.37 g) was statistically significantly greater [feeding condition: *F*_(1, 25)_ = 108.85, *p* < 0.0001] than the FDR group's BW (Sham: 310.17 ± 8.63 g; ADX: 312.00 ± 9.19 g). No statistically significant effects on BW for surgery group or the feeding condition × surgery group interaction were observed.

**Figure 6:**
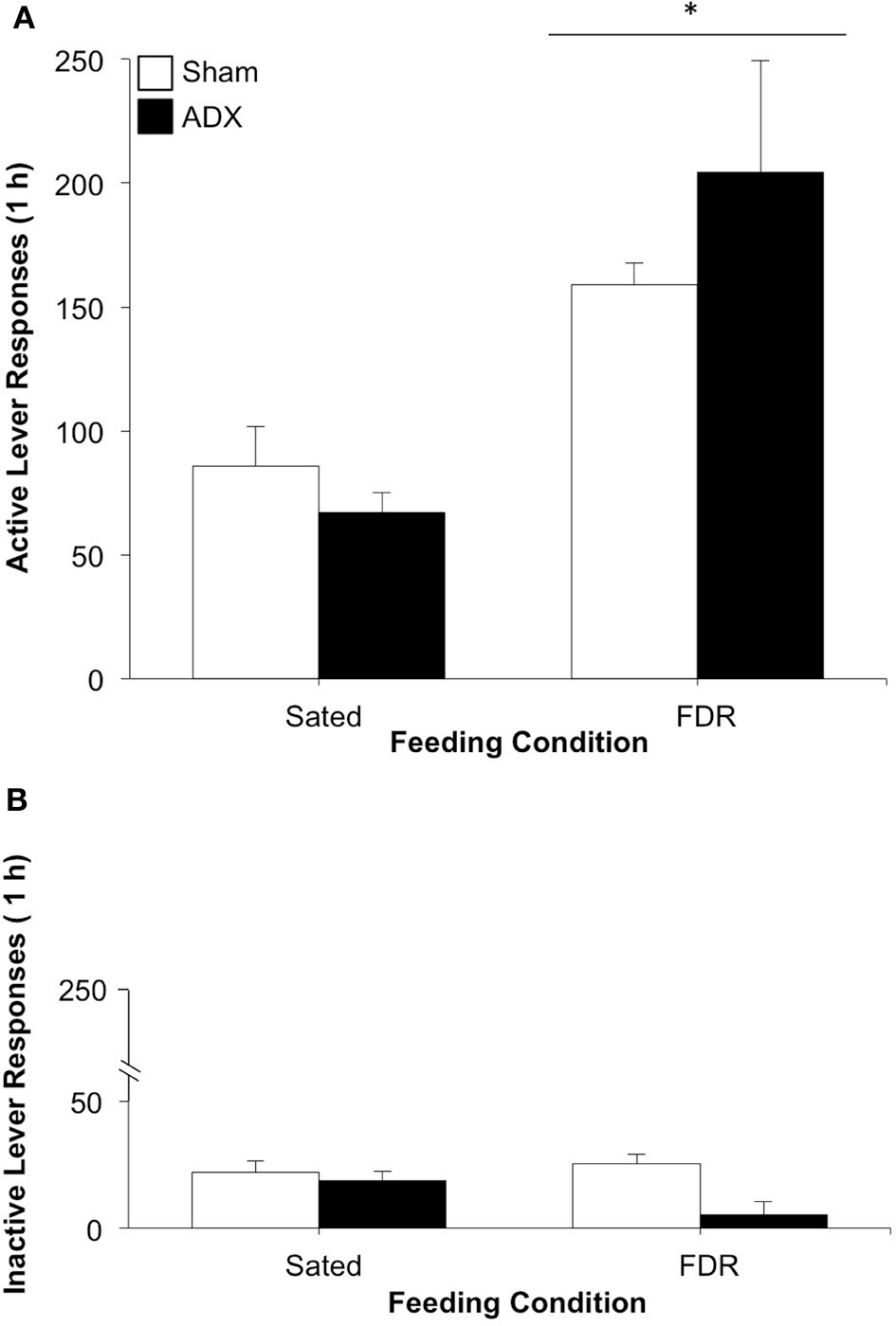
**The effect of adrenalectomy on heroin seeking in food-restricted (FDR) and sated rats under withdrawal; Experiment 2B.** Data shown are the mean (+SEM) numbers of active **(A)** and inactive **(B)** lever responses made on test day by rats in the FDR (*n*'s: FDR-SHAM = 6, FDR-ADX = 7) and Sated (*n*'s: Sated-SHAM = 7, Sated-ADX = 9). Test day consisted of 1-h drug-seeking session under extinction conditions, following heroin self-administration training and 16 days of withdrawal under FDR or sated conditions. ^*^Significantly different from the sated condition, *p* < 0.001.

As can be seen in Figure 6A, the FDR group pressed the active lever more than the Sated group [feeding condition effect: *F*_(1, 25)_ = 19.19, *p* < 0.001, η^2^ = 0.40] during the drug-seeking test. No statistically significant effects for surgery group or the feeding condition × surgery group interaction were observed. No significant effects were observed for inactive lever responding on test day (Figure 6B).

As expected, corticosterone levels in adrenalectomized rats were very low (<5 ng/ml), and were not affected by food restriction (data not shown).

## Discussion

Recent work in our laboratory has demonstrated an augmentation of heroin seeking in chronically food restricted rats, under withdrawal (D'Cunha et al., 2013). Thus, as expected, a prolonged period of food restriction resulted in a robust increase in heroin seeking, compared to sated rats, across all experimental groups in the current study. In contrast to the robust attenuation of acute food deprivation-induced reinstatement of drug seeking following treatment with CRF-receptor antagonists, treatment with R121919, a selective CRF_1_-R antagonist, or α-helical CRF, a non-specific CRF-R antagonist, did not result in a statistically significant reduction in heroin seeking in food restricted rats under withdrawal. Similarly, neither treatment with RU486, a glucocorticoid receptor antagonist, nor adrenalectomy affected heroin seeking in this model.

Our findings are consistent with considerable evidence supporting a modulatory role for food restriction on drug-related behaviors in humans (Hall et al., 1992; Krahn et al., 1992; Cheskin et al., 2005) and in laboratory animals, where food deficiency drastically influences drug taking and the reinforcing properties of abused drugs (Carroll and Meisch, 1984; Stuber et al., 2002; Carr, 2007).

Despite previous evidence demonstrating that CRF_1_-R antagonists attenuate acute food deprivation-induced reinstatement of extinguished cocaine and heroin seeking (Shalev et al., 2003, 2006), the experiments described here suggest that these findings do not extend to chronic food restriction-induced augmentation of heroin seeking in rats under withdrawal. The observed lack of effect for CRF-R antagonists in chronically food-restricted rats may be due to the differential effects of food restriction and deprivation on metabolism and behavior (Fulton et al., 2000) or differences between the reinstatement model and the withdrawal procedure used here. First, rats in the present study did not undergo a period of extinction. Extinction training and withdrawal (without extinction) result in activation of distinct neural circuits during drug-seeking tests (Fuchs et al., 2008), which might be differentially modulated by the CRF system. Second, our current study employed a prolonged period of mild stress induced by food restriction (Deroche et al., 1995) compared to the acute 24–48 h food deprivation we have used previously. Alterations in gene expression suggest that different neural adaptations occur following exposure to acute and chronic stress. For example, increased CRF_1_-R and c-fos mRNA in the paraventricular nucleus of the hypothalamus (PVN) are observed following acute, but not chronic stress. In contrast, chronic stress results in lowered levels of CRF_1_-R and c-fos mRNA in the PVN (Bonaz and Rivest, 1998). However, other reports have demonstrated the opposite result, where increased levels of CRF_1_-R mRNA in the PVN were reported following chronic but not acute stress (Imaki et al., 1991). Notwithstanding these inconsistencies, there appear to be distinct adaptations in the CRF system following exposure to acute or chronic stress.

In the present study, rats were exposed to a 14-day food restriction stress, which may have resulted in progressive CRF-induced adaptations in critical neuronal circuits over the withdrawal period. For example, greater dopamine (DA) tissue levels in the nucleus accumbens (NAc), and reduced levels in the prefrontal cortex (PFC), were found 1 week following the completion of a chronic treatment (13 days) with CRF (Izzo et al., 2005). Moreover, differential effects for acute vs. chronic treatment with CRF antagonists are suggested by findings of Mällo et al. (2004) who reported a reduction in anxiety (as defined by increased exploration) in an elevated-zero-plus-maze test following chronic, but not acute treatment with a selective CRF_1_-R antagonist. Consequently, acute CRF-R activation during the test may no longer be necessary to demonstrate the augmentation of heroin seeking in food restricted rats. Future studies should investigate the effects of chronic CRF-R antagonist treatment, over the withdrawal period, on the augmentation of heroin seeking induced by chronic food restriction.

An interesting, albeit not statistically significant, trend for a dose dependent reduction in responding on the previously heroin paired (active) lever on the test day was observed in the α-helical CRF-treated sated group. Recently, CRF-R antagonism was shown to reduce cue-induced reinstatement of drug seeking (Moffett and Goeders, 2006), which could provide a possible explanation for the reduction of active lever responding observed in sated rats in the current experiments, following exposure to the drug-associated environment and cues. However, a similar pattern was found for inactive lever responding in the food restricted and sated groups, suggesting that the reduced lever seeking in the α-helical CRF-treated rats was not due exclusively to changes in the motivational value of the drug-associated stimuli. Furthermore, administration of R121919 did not reduce active or inactive lever responding in the drug treated groups, further supporting a lack of motivational effects for CRF-R antagonists in the current procedure. It is possible that treatment with α-helical CRF resulted in an overall reduction of locomotor responding, which was obscured by the increased drug-seeking behavior in the food restricted rats; yet, we found no indication for such an effect in the previous studies conducted in our laboratory (Shalev et al., 2006).

To ensure the efficiency of the treatment with α-helical CRF, its effect on anxiety-related behaviors was investigated. In this test a reduction in anxiety was assessed by the latency to consume a food pellet placed in the center of an open field, and the number of approaches made prior to finally consuming the food. We found shorter latencies and reduced number of approaches made before food consumption in food restricted rats that received α-helical CRF treatment. Rats that did not receive the drug treatment approached the food multiple times with no attempt at consumption and would instead continue to explore the environment.

To gain a better understanding of the involvement of the physiological stress response in food restriction-induced augmentation of heroin seeking, we investigated the role of corticosterone, the major stress-associated hormone. Chronically food restricted rats exhibit greater levels of corticosterone compared to controls (Carr, 1996). Furthermore, the elevated concentrations observed following food restriction are positively associated with the proclivity to self-administer cocaine. Additionally, the removal of corticosterone via adrenalectomy can also decrease the psychostimulant challenge-induced heightened locomotor activity observed in food restricted rats (Deroche et al., 1995; Piazza and Le Moal, 1996). It can be argued that the lack of effect following treatment with CRF-R antagonists indicates that the complete HPA-axis is not involved in food restriction-induced augmentation of heroin seeking. However, plasma levels of adrenocorticotropic hormone (ACTH) and the subsequent production of corticosterone can be affected by mechanisms independent of CRF's actions in the HPA axis (Tsigos and Chrousos, 2002).

In the current study, neither acute treatment with a glucocorticoid antagonist, RU486, nor adrenalectomy reduced the increased heroin seeking observed in chronically food-restricted rats under withdrawal. These results are consistent with past studies in the literature on stress- and reward-related behaviors. In CRF deficient mice, activity in an anxiety provoking situation (e.g., elevated plus maze) remains unaffected, in spite of a blunted HPA axis response and lowered concentrations of corticosterone (Dunn and Swiergiel, 1999). Therefore, a heightened physiological stress response may not always be necessary for the expression of stress-related behaviors. Abrahamsen and Carr (1996) demonstrated that in a lateral hypothalamus self-stimulation procedure, the sensitization of the rewarding effects of the stimulation by food restriction is unaltered following a treatment with a corticosterone synthesis inhibitor or an acute feeding-induced decrease in plasma corticosterone (Abrahamsen et al., 1995). The aforementioned studies argue against a modulatory role for corticosterone in rewarding-seeking. As Carr (2002) suggests, however, the most comprehensive test of corticosterone's involvement in food restriction would be to maintain corticosterone concentrations in the food restricted group at similar concentrations as those reported in the sated controls over the full period of restriction. A recent study from DiLeone's group (Guarnieri et al., 2012) offers further support for a mediating role for corticosterone in the effects of food restriction on motivation. Guarnieri et al. (2012) report that 5 days of mild food restriction in mice resulted in an upregulation of genes in the mesocorticolimbic system that are associated with the stress response. These changes in gene expression were critically dependent on food restriction-induced increases in corticosterone levels, and the hormone was also shown to be important for the potentiation of food seeking in food restricted mice. The authors suggest that the identified genes are the molecular signals that drive food restriction-induced behavioral plasticity (Guarnieri et al., 2012). It is important to note, however, that some of these corticosterone-dependent changes in gene expression were triggered following only 1 day of food restriction. In contrast, we have reported that a short-term food restriction period (3–5 days) is not sufficient to induced augmentation of heroin seeking (D'Cunha et al., 2013), suggesting that different, slower to develop, adaptations underlie this phenomenon.

Concentrations of corticosterone were statistically significantly greater in food restricted and sated rats after treatment with RU486 compared to the vehicle pretreatment. Interestingly, the magnitude of increase in the food-restricted group (~500%) was greater than that in the sated group (~74%) following RU486 treatment. We speculate that these differences in magnitude can be explained by the lack of negative feedback from circulating corticosterone, which resulted in amplification of the increased corticosterone levels that are typically found in food restricted rats.

There is evidence that the augmentation of drug seeking following food restriction can be modulated by homeostatic mechanisms that are triggered by the hunger state (Cabeza de Vaca and Carr, 1998). For example, infusions of ghrelin, an orexigenic gut hormone, can increase extracellular DA concentrations in the NAc (Jerlhag et al., 2007), and cocaine-induced increases of extracellular DA in the NAc are attenuated by ghrelin receptor antagonism (Jerlhag et al., 2010). Importantly, increased serum concentrations of ghrelin have been observed in response to cue-induced reinstatement of cocaine seeking (Tessari et al., 2007), suggesting an involvement of the peptide in the conditioned reinforcing effects of cocaine. However, the involvement of ghrelin in cocaine seeking and taking might not be easily generalized to other drugs. Treatment with a ghrelin antagonist did not impair food deprivation-induced reinstatement of heroin seeking, although central infusions of ghrelin did increase the breakpoints on a progressive ratio schedule of heroin reinforcement (Maric et al., 2011). It is critical to note that the aforementioned study used acute food deprivation in a reinstatement of extinguished drug seeking procedure, which as mentioned above, might involve different brain mechanisms than prolonged food restriction in rats under withdrawal.

An additional peripheral signal that is involved in energy balance is leptin, an anorexigenic hormone that is secreted by peripheral adipocytes (Friedman and Halaas, 1998). Leptin can regulate activity in the mesocorticolimbic circuitry through its actions on ventral tegmental area (VTA) DA neurons, and has been implicated in reward processes (Fulton et al., 2000). Interestingly, leptin was shown to attenuate acute food deprivation-induced reinstatement of heroin seeking (Shalev et al., 2001). However, this effect was not observed with footshock stress- or heroin priming-induced reinstatement (Shalev et al., 2001), suggesting that leptin's effect on acute food deprivation-induced reinstatement was not mediated by DA or stress-related pathways. In contrast, it is possible that in chronically food-restricted rats, as in the current study, the decrease in leptin signal contributed to the sensitized response to the drug-associated cues through disinhibition of the mesocorticolimbic DA system (Hommel et al., 2006).

In conclusion, we suggest that pathways involved in the acute stress response are not critical for the expression of augmented drug seeking in food-restricted rats under withdrawal. Our findings, however, do not exclude a role for food restriction-induced prolonged increases in CRF and corticosterone in the induction of downstream brain adaptations, which in turn may drive the augmented drug seeking observed in rats under withdrawal from heroin.

### Conflict of interest statement

The authors declare that the research was conducted in the absence of any commercial or financial relationships that could be construed as a potential conflict of interest.
